# Influenza vaccine hesitancy and influencing factors among university students in China: a multicenter cross-sectional survey

**DOI:** 10.1080/07853890.2023.2195206

**Published:** 2023-04-27

**Authors:** Haiyan Zou, Yan Huang, Ting Chen, Luying Zhang

**Affiliations:** a School of Public Health, Fudan University, Shanghai, China; b Center for Chinese Public Administration Research, School of Government, Sun Yat-sen University, Guangzhou, China; c School of Public Health, Wuhan University of Science and Technology, Wuhan, China

**Keywords:** Influenza vaccine, vaccine hesitancy, hesitancy matrix, influencing factors, university students

## Abstract

**Aim:**

Highly mutable and contagious influenza poses a serious health threat to university students and their close contacts. Although annual influenza vaccination is an effective way to prevent influenza, influenza vaccination rates among Chinese university students are still low due to vaccine hesitancy. This study investigated Chinese university students’ hesitancy to receive influenza vaccine and its influencing factors during the COVID-19 pandemics based on WHO’s vaccine hesitancy matrix.

**Methods:**

A multicenter cross-sectional study of university students in four cities across China was conducted via a web-based questionnaire in June 2022. Binary logistic regression was adopted to determine the factors around contextual influences, individual and group influences, and vaccines/vaccination specific issues. The reliability and validity of the questionnaire were good, with a Kronbach alpha coefficient of 0.892 and a KMO coefficient of 0.957.

**Results:**

Of the 2261 Chinese university students surveyed, 44.7% had influenza vaccine hesitancy. Binary logistic regression showed that students considering high severity (OR = 0.946) or probability (OR = 0.942) of getting influenza, trusting vaccine-related advice from medical personnel (OR = 0.495) had lower odds of hesitancy. The odds of influenza vaccine hesitancy were higher if the students believed that vaccination was not necessary (OR = 4.040), had not been recommended by people around (OR = 1.476) and had no previous vaccinations or appointments (OR = 2.685).

**Conclusions:**

Medical staff are suggested to provide health education, improve doctor-patient communication and recommend vaccinations to university students to increase their risk perception and willingness to get an influenza vaccination. Collective vaccination strategies can be implemented to reduce the vaccine hesitancy for students.

## 1. Introduction

Influenza is an acute respiratory disease caused by an influenza virus. Influenza virus is characterized by strong variability, rapid transmission and high infectivity. It can occur in a large scale in a particular season and in crowded places such as schools. The influenza incidence rate in mainland China in 2018 was 5.51 per 10,000 people [1], and caused a huge disease economic burden of 26.38 billion CNY in 2019, of which the hospitalization-related accounted for 86.4% [2].

Annual influenza vaccination is an effective way to prevent influenza and can reduce the risk of influenza and related complications [3–5]. Although anyone can be susceptible to influenza, the coverage rate of influenza vaccine in China is still low. In 2021–2022, the total influenza vaccine coverage rate in China was 2.47% [6], much lower than many countries such as the United States (51.40%) [7], Brazil (57.35%) [4] and England (48.0%) [8].

There is a gap between actual influenza vaccination rates and the probability of being willing to receive an influenza vaccine. Vaccine hesitancy refers to delay in acceptance or refusal of vaccination despite availability of vaccination services [9], and has been listed by the WHO as one of the top 10 health threats [10]. Vaccine hesitancy may affect public confidence and acceptance of vaccines, reduce vaccination rates and herd immunity, and increase the likelihood of preventable disease outbreaks and epidemics [11,12]. A decline in the influenza vaccination rate due to vaccine hesitancy has been reported in many countries in recent years, such as in the United States [13], France [14] and Canada [15]. Therefore, in order to improve vaccination acceptance, it is necessary to study the influencing factors of influenza vaccine hesitancy.

Highly contagious influenza poses a serious health threat to university students and their close contacts [16]. On the one hand, flu headaches, cough and other symptoms can affect students’ class performance or extracurricular activities [17]. On the other hand, due to the dense living space and frequent social activities, influenza can easily spread on campus, posing a major threat to the health of campus students [18]. Besides, influenza can also spread from students to vulnerable family members or community members, such as the elderly and children [19]. Therefore, it is necessary to investigate the influenza vaccine hesitancy and the influencing factors among the university student population.

Few studies focused on influenza vaccination among Chinese university students. One study investigated the prevalence and factors of influenza vaccination among Chinese university students, based on the Health Belief Model [20]. Another looked at the impact of different sources of information on influenza vaccination [21]. The rest of the study have mainly focused on children or their parents [22,23], the elderly [24], medical students [25] and medical personnel’s [26].

To explore the determinants of vaccine hesitancy, WHO Strategic Advisory Group of Experts on Immunization (SAGE) constructed a vaccine hesitancy matrix, including contextual influences, individual and group influences, and vaccine/vaccination-specific issues [27]. In this study, we used the vaccine hesitancy matrix to design a questionnaire to study the hesitancy and its influencing factors of influenza vaccine among Chinese university students, so as to provide reference for the study on influenza vaccine hesitancy and suggestions for the customized vaccine plan for university students in China.

## 2. Methods

### 2.1. Study design

This study used a multicenter cross-sectional and survey-based research methodology. The independent variables in the study were based on the vaccine hesitancy matrix, while the dependent variable was whether or not to hesitate to get the influenza vaccine. Inclusion criteria for participants in this study were: (1) current enrolment at sampling universities in Shanghai, Wuhan, Guangzhou and Nanning; (2) having access to the Internet *via* computer or smart phone; (3) providing informed consent. This study was ethically reviewed and approved by the Institutional Review Board, School of Public Health, Fudan University (IRB#2022-08-0992).

### 2.2. Survey instruments

We used the vaccine hesitancy matrix to design the questionnaire (Table 1). Contextual influences include historic, socio-cultural, environmental, economic or political factors. Individual and group influences arise from personal perception of the vaccine or influences of the social/peer environment. Vaccine/vaccination-specific issues are directly related to vaccine or vaccination. The degree of influenza vaccine hesitancy was indicated on a scale of 0–10 (0 representing vaccine acceptance, 1–10 representing hesitancy). The Kronbach alpha coefficient for the scale was 0.892 and the KMO coefficient was 0.957. This indicated that the reliability and validity of the scale were good. A pilot survey was conducted with 60 students for testing readability and logic of the questionnaire. And it was proved to be easily understood and clearly expression.

**Table 1.** Questions used to measure influencing factors in survey instrument.

Dimension	Questions	Question design
Contextual influences	Gender	1 = male; 2 = female
	Age	1 = Less than or equal to 21 years; 2 = Older than 21 years
	City	1 = Shanghai; 2 = Wuhan; 3 = Guangzhou; 4 = Nanning
	Major	1 = Science and technology or agriculture; 2 = Social Sciences; 3 = Medicine
	Education level	1 = Junior college; 2 = Undergraduate; 3 = Postgraduate
	Residence	1 = Rural; 2 = Urban
	Are you insured for basic medical insurance?	1 = Yes; 2 = No
	What are your monthly living expenses (CNY)?	1 = Less than 1000; 2 = 1001–2000; 3 = More than 2000
	Do you have a family history of cancer?	1 = Yes; 2 = No
	Have you ever had influenza?	1 = Yes; 2 = No
	Have you heard the negative information about vaccines?	1 = Yes; 2 = NO
Individual and group influences	How much do you know about the influenza vaccine?	0–4 (add up the scores of the correct options)
	What do you think of the severity of being infected by influenza?	0–10 (‘very low’ to ‘very high’)
	What do you think of the probability of being infected by influenza?	0–10 (‘very low’ to ‘very high’)
	How do you fear of being infected by influenza?	0–10 (‘very low’ to ‘very high’)
	What do you think of the necessity of influenza vaccination?	1 = Yes; 2 = No
	Do you trust the vaccine-related advice given by medical professionals?	1 = Yes; 2 = No
	Have you been recommended by your family, classmates or friends to the influenza vaccine?	1 = Yes; 2 = No
vaccine/vaccination-specific issues	Do you believe the efficacy of vaccines?	1 = Yes; 2 = No
	Do you believe the safety of domestic vaccines?	1 = Yes; 2 = No
	Do you believe the safety of vaccines abroad?	1 = Yes; 2 = No
	Have you been recommended by your doctor to the influenza vaccine?	1 = Yes; 2 = No
	Have you been vaccinated against COVID-19?	1 = Yes; 2 = No
	Have you ever had an influenza vaccination/appointment?	1 = vaccination; 2 = appointment; 3 = neither

### 2.3. Sample size and data collection

In this study, we used a stratified sampling method. The sampling cities, one municipal city (Shanghai) and three provincial capitals in the east, middle and west regions (Guangzhou, Wuhan and Nanning), were selected as the sampling cities, taking into account the geographical location and socioeconomic development level. In each city, the study group selected 10 to 20 universities, covering different levels of education (college, undergraduate, graduate) and majors (science, technology, social sciences, medicine). Then, two classes were randomly selected in each sample college and the link or QR code of the web questionnaire was sent to the college students through the lecturer to fill in. Data was collected between June 1st and 30th 2022 and was supported by www.wjx.cn. Respondents would only start answering the questionnaire after confirming the informed consent button. Only 1 submission per account was allowed. After the web-based questionnaires were collected, the quality control personnel eliminated the following questionnaires: (1) the questionnaire response time was too short (less than 180 s); (2) the questionnaire responses were logically contradictory or incorrect.

A priori sample size per group was estimated by the following formula based on 5% type one error:

$$n=\frac{Z_{1\text{-}\alpha /2}^{2}\times p(1-p)}{\delta^{2}}$$

In this study, the willingness rate of influenza vaccine was based on the research results of Jiang et al. [28], which found that from 45.0% to 53.1% of general population adults in China expressed willingness to receive influenza vaccine. Therefore, this study estimated the willingness rate of influenza vaccine(p) to be about 50%, and maximum permissible error (δ)= 0.1p. The minimum sample size per city *n* = 385 was calculated. Considering the risk of bias in online surveys, this study appropriately expanded the sample size by 30%, resulting in *n* = 500 in each city.

### 2.4. Statistical analysis

The EXCEL data exported from www.wjx.cn was processed and analyzed using spss25.0 software. First, we described the basic characteristics of the survey respondents, the frequency and percentage of vaccine hesitancy. Second, we applied the chi-square test for comparison. Finally, a binary logistic regression model was adopted to compare the two categories of vaccine hesitancy or vaccine non-hesitancy. The results were presented with odds ratios (ORs) and 95% confidence intervals (CIs). The level of statistical significance was a *p*-value < .05.

## 3. Results

### 3.1. Demographic characteristics of participants

A total of 2261 valid questionnaires were collected in this survey. As shown in Table 2, the survey respondents were mainly female, accounting for 63.6%. The age was mainly beyond 21 years old, accounting for 70.0%. The proportion of junior college, undergraduates and postgraduates were 20.0%, 66.3% and 13.7% respectively. Overall, 42.5% and 41.2% of participants majored in medicine and social science respectively. Among the surveyed population, 53.7% of university students lived in rural areas, 93.0% participated in basic medical insurance, 59% lived on 1001–2000 CNY (144.5- 288.7 USD) per month, and 91.3% had no family history of cancer.

**Table 2.** Participants’ characteristics and attitudes toward influenza vaccines.

Factors		Total, *n* (%)	Attitude to vaccination, *n* (%)		*p* value
			No hesitancy	Hesitancy	
Gender	male	823 (36.4)	422 (51.3)	401 (48.7)	.004
	female	1438 (63.6)	828 (57.6)	610 (42.4)	
Age (years)	≤21	1583 (70.0)	923 (58.3)	660 (41.7)	<.001
	>21	678 (30.0)	327 (48.2)	351 (51.8)	
City	Shanghai	687 (30.4)	413 (60.1)	274 (39.9)	<.001
	Wuhan	548 (24.2)	290 (52.9)	258 (47.1)	
	Guangzhou	488 (21.6)	220 (45.1)	268 (54.9)	
	Nanning	538 (23.8)	327 (60.8)	211 (39.2)	
Education level	Junior college	452 (20.0)	302 (66.8)	150 (33.2)	<.001
	Undergraduate	1499 (66.3)	819 (54.6)	680 (45.4)	
	Postgraduate	310 (13.7)	129 (41.6)	181 (58.4)	
Major	Science and technology/ Agriculture	368 (16.3)	210 (57.1)	158 (42.9)	.010
	Social sciences	932 (41.2)	544 (58.4)	388 (41.6)	
	Medicine	961 (42.5)	496 (51.6)	465 (48.4)	
Residence	Rural	1214 (53.7)	697 (57.4)	517 (42.6)	.028
	Urban	1047 (46.3)	553 (52.8)	494 (47.2)	
Medical insurance	Yes	2102 (93.0)	1156 (55.0)	946 (45.0)	.313
	No	159 (7.0)	94 (59.1)	65 (40.9)	
Living expense (CNY)	<1000	294 (13.0)	177 (60.2)	117 (39.8)	.154
	1001–2000	1440 (63.7)	792 (55.0)	648 (45.0)	
	>2000	527 (23.3)	281 (53.3)	246 (46.7)	
Family history of cancer	Yes	196 (8.7)	83 (42.3)	113 (57.7)	<.001
	No	2065 (91.3)	1167 (56.5)	898 (43.5)	

### 3.2. Vaccine hesitancy

Of the 2261 Chinese university students surveyed, 1250 (55.3%) were not vaccine hesitant while 1011 (44.7%) had vaccine hesitancy. Table 2 showed that the sociodemographic characteristics of university students were significantly different (*p* < .05) from vaccination hesitancy in terms of gender, age, city, education level, major, residence, and family history of cancer.

### 3.3. Knowledge, perceptions, attitudes and behaviours toward influenza vaccination

In this section, we explored the barriers and facilitators of vaccine hesitancy among university students to propose influenza vaccination strategies. 8% of university students had ever got the influenza and 34.6% of students heard the negative information about vaccines. The mean scores for severity, probability and fear of being infected by influenza were 5.67, 4.48 and 4.17 respectively. 66.9% agreed that influenza vaccination was necessary and 76.1% believed in the efficacy of vaccines. A small number of students (17.9%) were well informed about the influenza vaccine and 33.1% were recommended for vaccines by those around them. Most students (72.2%) trusted the recommendations of medical personnel for vaccines, but only a small percentage (30.8%) had ever received a doctor’s recommendation. 64% of students trusted the safety of domestic vaccines while 59% trusted vaccines abroad. Almost everyone (99%) had been vaccinated against the COVID-19 and only 33.8% had ever injected influenza vaccines. The vast majority of variables in Table 3 were significantly related to vaccine hesitancy (*p* < .05).

**Table 3.** Influencing factors associated with influenza vaccine hesitation.

Matrix	Factors		Total, *n* (%)/Average, $\bar{X}$	Attitude to vaccination		*p*
				No hesitancy	Hesitancy	
Contextual influences	Have ever got influenza	Yes	181 (8.0)	92 (50.8)	89 (49.2)	.209
		No	2080 (92.0)	1158 (55.7)	922 (44.3)	
	The negative information about vaccines	Yes	783 (34.6)	483 (61.7)	300 (38.3)	<.001
		No	1478 (65.4)	767 (51.9)	711 (48.1)	
Individual and group influences	Vaccine knowledge	low	867 (38.3)	426 (49.1)	441 (50.9)	<.001
		moderate	990 (43.8)	583 (58.9)	407 (41.1)	
		high	404 (17.9)	241 (59.7)	163 (40.3)	
	The severity of being infected by influenza	0–10	5.67	6.09	5.14	<.001
	The probability of being infected by influenza	0–10	4.48	4.80	4.09	<.001
	The fear of being infected by influenza	0–10	4.17	4.57	3.68	<.001
	The necessity of influenza vaccination	Yes	1513 (66.9)	1065 (70.4)	448 (29.6)	<.001
		No	748 (33.1)	185 (24.7)	563 (75.3)	
	Vaccines recommended by people around	Yes	749 (33.1)	562 (75.0)	187 (25.0)	<.001
		No	1512 (66.9)	688 (45.5)	824 (54.5)	
	Trust vaccine-related advice provided by medical staffs	Yes	1632 (72.2)	1050 (64.3)	582 (35.7)	<.001
		No	629 (27.8)	200 (31.8)	429 (68.2)	
Vaccine/vaccination-specific issues	The efficacy of vaccines	Yes	1720 (76.1)	1064 (61.9)	656 (38.1)	<.001
		No	541 (23.9)	186 (34.4)	355 (65.6)	
	The safety of domestic vaccines	Yes	1446 (64.0)	(62.0)	550 (38.0)	<.001
		No	815 (36.0)	354 (43.4)	461 (56.6)	
	The safety of vaccines abroad	Yes	1334 (59.0)	829 (62.1)	505 (37.9)	<.001
		No	927 (41.0)	421 (45.4)	506 (54.6)	
	Influenza vaccines recommended by doctor	Yes	697 (30.8)	505 (72.5)	192 (27.5)	<.001
		No	1564 (69.2)	745 (47.6)	819 (52.4)	
	Have been vaccinated against COVID-19	Yes	2238 (99.0)	1243 (55.5)	995 (44.5)	.016
		No	23 (1.0)	7 (30.4)	16 (69.6)	
	Have an influenza vaccination/appointment	Vaccination	765 (33.8)	599 (78.3)	166 (21.7)	<.001
		Appointment	84 (3.7)	40 (47.6)	44 (52.4)	
		Neither	1412 (62.5)	611 (43.3)	801 (56.7)	

The result of binary logistic regression has been shown in Table 4. The possibility of influenza vaccine hesitancy was lower for students who considered the severity (OR = 0.946, CI: 0.901–0.993) or probability (OR = 0.942, CI: 0.894–0.993) of contracting influenza to be high or trusted vaccine-related advice provided by medical staffs (OR = 0.495, CI: 0.380–0.644). The possibility of influenza vaccine hesitancy was higher if the students believed that vaccination was not necessary (OR = 4.040, CI: 3.124–5.225), had not been recommended by people around (OR = 1.476, CI: 1.088–2.002) and had no previous vaccinations or appointments (OR = 2.685, CI: 2.097–3.439). Besides, female students (OR = 0.662, CI: 0.535–0.820) or students with no family history of cancer (OR = 0.691, CI: 0.486–0.982) were less likely to hesitate to get vaccine while those who had an undergraduate (OR = 1.672, CI: 1.170–2.390) or bachelor’s degree (OR = 2.387, CI: 1.460–3.902) were more hesitant.

**Table 4.** Binary logistic regression to identify factors associated with influenza vaccine hesitancy.

Matrix	Factors		Hesitancy vs. No hesitancy			
			OR	*p*	95% CI	
Contextual influences	Gender	(Rf: male)				
		Female	0.662	<.001	0.535	0.820
	Education Level	(Rf: Junior college)				
		Undergraduate	1.672	.005	1.170	2.390
		Postgraduate	2.387	.001	1.460	3.902
	Family history of cancer	(Ref: Yes)				
		No	0.691	.039	0.486	0.982
Individual and group influences	The severity of being infected by influenza		0.946	.025	0.901	0.993
	The probability of being infected by influenza		0.942	.026	0.894	0.993
	The necessity of influenza vaccination	(Rf: Yes)				
		No	4.040	<.001	3.124	5.225
	vaccines recommended by people around	(Rf: Yes)				
		No	1.476	.012	1.088	2.002
	Trust vaccine-related advice provided by medical staffs	(Rf: No)				
		Yes	0.495	<.001	0.380	0.644
vaccine/vaccination-specific issues	Have a influenza vaccination /appointment	(Rf: vaccination)				
		Appointment	3.604	<.001	2.158	6.019
		Neither	2.685	<.001	2.097	3.439

## 4. Discussion

This study analyzed the current situation of influenza vaccine hesitancy and its influencing factors among university students in four cities across China, based on the vaccine hesitancy matrix proposed by the World Health Organization. The survey results showed that the vaccine hesitancy rate was 44.7%. This result was consistent with previous findings examining influenza vaccine hesitancy in the general population in China [29].

This study found that three dimensions had an impact on university students getting the influenza vaccine. The first were contextual influences, such as sociodemographic characteristics. The second were individual and group influences, such as personal risk perception, trust in medical personnel and influence of people around. The third were vaccine/vaccination specific issues, such as personal vaccination experience.

In terms of individual and group influences, personal risk perception and trust in medical personnel are significant influencing factors. On the one hand, risk perception was an individual’s subjective judgment of disease susceptibility. It included perceived severity, necessity and probability of getting influenza. There was a significant consistency between risk perception and vaccination behaviour [30,31]. Lack of knowledge or misconceptions about influenza and influenza vaccine could affect students’ personal risk perception and willingness to receive vaccination. On the other hand, medical personnel were the most trusted source of vaccination information for most university students, and trust between them was the cornerstone for maintaining confidence in vaccination. Medical personnel’s knowledge of and attitudes toward the vaccine have been proven to be important determinants of their own vaccination and their recommendation of the vaccine to their patients [32]. Therefore, it is recommended that medical staff provide health education, improve doctor-patient communication and recommend vaccinations to university students in order to further increase the level of risk perception and reduce vaccine hesitancy among students.

In addition, chi-square tests and logistic regressions indicated that college students were more likely to get vaccinated if they had been recommended for the influenza vaccine by people close to them, such as family, classmates and friends. In general, college students’ daily life trajectory was mainly at school and home, and their awareness of diseases and preventative immunization behaviors were strongly influenced by those around them. This suggested that collective vaccination by class, school or community may be more effective than individual vaccination for university students [33]. For example, the medical college of Wisconsin COVID-19 Vaccination Program aimed to support state public health agencies in providing access to vaccination for underserved and higher education community members [34]. Collective vaccination strategy may have two major benefits. Firstly, it has improved the convenience of vaccination [35]. Collective vaccination means uniform appointments and a fixed time and place for vaccination, making it much more convenient for busy students. Secondly, it can increase students’ sense of collective responsibility [36] and thus make them reduce the vaccine hesitancy.

In terms of personal vaccination experience, university students who had received the influenza vaccine before were more likely to continue receiving the vaccine. This was consistent with previous studies. Besides, the study found that gender, education level, and family history of cancer would influence the university students’ willingness to receive influenza vaccination, which was found in other studies as well [37].

To our knowledge, this was the first investigation of the current status of influenza vaccination hesitancy and its influencing factors among Chinese university students based on the vaccine hesitancy matrix, especially during the Covid-19 pandemic. A total of 2,261 valid questionnaires were collected from universities across Shanghai, Guangzhou, Wuhan and Nanning. By using the multi-stage stratified sampling and conducting a multi-center survey, the study had an adequate sample size and good representation. The findings can be used widely. They were not only useful for the development of influenza vaccination strategies, but also had implications for the vaccination strategies of other self-funded vaccines. However, there were some limitations in this study. The study was a cross-sectional survey, and causal inference could not be examined. In addition, the study used a web-based questionnaire, which may be subject to sampling bias.

## 5. Conclusion

This study investigated the hesitancy of Chinese university students to receive influenza vaccination by conducting a multi-center cross-sectional questionnaire survey and found that 44.7% of students were vaccine hesitant. Based on the vaccine hesitancy matrix, this study found that the factors influencing Chinese college students’ vaccine hesitancy were risk perceptions of the severity of influenza, the likelihood of getting influenza, and the necessity of vaccination, trusting the recommendations of medical personnel, being recommended for vaccination by people close to them, and having been vaccinated against influenza before. Therefore, medical staff are suggested to provide health education, improve doctor-patient communication and recommend vaccinations to university students to increase their risk perception and willingness to get an influenza vaccination. Collective vaccination strategies can be implemented to reduce the vaccine hesitancy for students.

## Data Availability

Not applicable.
